# REViewer: haplotype-resolved visualization of read alignments in and around tandem repeats

**DOI:** 10.1186/s13073-022-01085-z

**Published:** 2022-08-11

**Authors:** Egor Dolzhenko, Ben Weisburd, Kristina Ibañez, Indhu-Shree Rajan-Babu, Christine Anyansi, Mark F. Bennett, Kimberley Billingsley, Ashley Carroll, Samuel Clamons, Matt C. Danzi, Viraj Deshpande, Jinhui Ding, Sarah Fazal, Andreas Halman, Bharati Jadhav, Yunjiang Qiu, Phillip A. Richmond, Christopher T. Saunders, Konrad Scheffler, Joke J. F. A. van Vugt, Ramona R. A. J. Zwamborn, Genomics England Research Consortium, Samuel S. Chong, Jan M. Friedman, Arianna Tucci, Heidi L. Rehm, Michael A. Eberle

**Affiliations:** 1 Illumina Inc., San Diego, CA 92122 USA; 2 Program in Medical and Population Genetics, Broad Institute of MIT and Harvard, Cambridge, USA; 3 Center for Genomic Medicine, Massachusetts General Hospital, Boston, USA; 4 William Harvey Research Institute, Queen Mary University of London, London, EC1M 6BQ UK; 5 Department of Medical Genetics, University of British Columbia and Children’s & Women’s Hospital, Vancouver, BC V6H3N1 Canada; 6 Department of Medical and Molecular Genetics, King’s College London, Strand, London, WC2R 2LS UK; 7 Population Health and Immunity Division, The Walter and Eliza Hall Institute of Medical Research, Parkville, VIC 3052 Australia; 8 Department of Medical Biology, University of Melbourne, Parkville, VIC 3052 Australia; 9 Epilepsy Research Centre, Department of Medicine, University of Melbourne, Austin Health, Heidelberg, VIC 3084 Australia; 10 Center for Alzheimer’s and Related Dementias, National Institute on Aging, Bethesda, MD USA; 11 Laboratory of Neurogenetics, National Institute on Aging, Bethesda, MD USA; 12 Dr. John T. Macdonald Foundation Department of Human Genetics and John P. Hussman Institute for Human Genomics, University of Miami, Miller School of Medicine, Miami, FL 33136 USA; 13 Computational Biology Group, Laboratory of Neurogenetics, National Institute on Aging, NIH, Bethesda, MD 20892 USA; 14 Peter MacCallum Cancer Centre, Melbourne, VIC 3000 Australia; 15 Sir Peter MacCallum Department of Oncology, The University of Melbourne, Parkville, VIC 3010 Australia; 16 Department of Genetics and Genomic Sciences and Mindich Child Health and Development Institute, Icahn School of Medicine at Mount Sinai, New York, NY 10029 USA; 17 BC Children’s Hospital Research Institute, Vancouver, BC V5Z 4H4 Canada; 18 Department of Neurology, University Medical Center Utrecht Brain Center, Utrecht University, Utrecht, The Netherlands; 19 Genomics England, Queen Mary University of London, Charterhouse Square, London, EC1M 6BQ UK; 20 Department of Pediatrics, Yong Loo Lin School of Medicine, National University of Singapore, Singapore, 119228 Singapore; 21 Department of Obstetrics and Gynecology, Yong Loo Lin School of Medicine, National University of Singapore, Singapore, 119228 Singapore; 22 Department of Laboratory Medicine, National University Hospital, Singapore, 119074 Singapore

**Keywords:** Repeat expansions, Short tandem repeats, Visualization, Short-read sequencing data

## Abstract

**Background:**

Expansions of short tandem repeats are the cause of many neurogenetic disorders including familial amyotrophic lateral sclerosis, Huntington disease, and many others. Multiple methods have been recently developed that can identify repeat expansions in whole genome or exome sequencing data. Despite the widely recognized need for visual assessment of variant calls in clinical settings, current computational tools lack the ability to produce such visualizations for repeat expansions. Expanded repeats are difficult to visualize because they correspond to large insertions relative to the reference genome and involve many misaligning and ambiguously aligning reads.

**Results:**

We implemented REViewer, a computational method for visualization of sequencing data in genomic regions containing long repeat expansions and FlipBook, a companion image viewer designed for manual curation of large collections of REViewer images. To generate a read pileup, REViewer reconstructs local haplotype sequences and distributes reads to these haplotypes in a way that is most consistent with the fragment lengths and evenness of read coverage. To create appropriate training materials for onboarding new users, we performed a concordance study involving 12 scientists involved in short tandem repeat research. We used the results of this study to create a user guide that describes the basic principles of using REViewer as well as a guide to the typical features of read pileups that correspond to low confidence repeat genotype calls. Additionally, we demonstrated that REViewer can be used to annotate clinically relevant repeat interruptions by comparing visual assessment results of 44 *FMR1* repeat alleles with the results of triplet repeat primed PCR. For 38 of these alleles, the results of visual assessment were consistent with triplet repeat primed PCR.

**Conclusions:**

Read pileup plots generated by REViewer offer an intuitive way to visualize sequencing data in regions containing long repeat expansions. Laboratories can use REViewer and FlipBook to assess the quality of repeat genotype calls as well as to visually detect interruptions or other imperfections in the repeat sequence and the surrounding flanking regions. REViewer and FlipBook are available under open-source licenses at https://github.com/illumina/REViewer and https://github.com/broadinstitute/flipbook respectively.

**Supplementary Information:**

The online version contains supplementary material available at 10.1186/s13073-022-01085-z.

## Background

Visual inspection of sequencing data supporting a given genetic variant is an important part of clinical bioinformatics pipelines. Effective visualizations enable scientists to quickly assess the quality of sequencing data supporting a genotype call. Factors that impact genotyping accuracy such as local depth, evenness of coverage, presence of any additional variation, and other locus-specific features are difficult to piece together from genome-wide quality metrics and various per-variant scores typically reported by variant calling methods. Recent guidelines from the Association for Medical Pathology and the College of American Pathologists strongly recommend review of such visualizations during routine sign out of variant calls [1].

The Integrative Genomics Viewer [2], JBrowse [3], and other general-purpose tools for visualization of sequencing data work well for single nucleotide variants, short indels, and copy number variants. Additionally, specialized methods have been developed for visualizing reads associated with variants that involve more complex indel patterns and distal breakpoints [4–7]. However, there is a lack of methods for visualizing sequencing data in regions harboring long repetitive sequences such as long stretches of short tandem repeats (STRs).

Analysis and visualization of regions containing long STRs using short read sequencing data pose a number of unique challenges. For instance, it is difficult to correctly align reads originating within the sequence of a long STR because the number of possible alignment positions increases linearly with the length of the STR allele. Regions containing multiple adjacent STRs—including the regions linked with Huntington disease, Friedreich ataxia, and Spinocerebellar ataxia 8—are especially prone to alignment artifacts because adjacent repeats may have a high sequence similarity and because the sizes of these repeats in a given individual often differ from those in the reference genome.

**Fig. 1:**
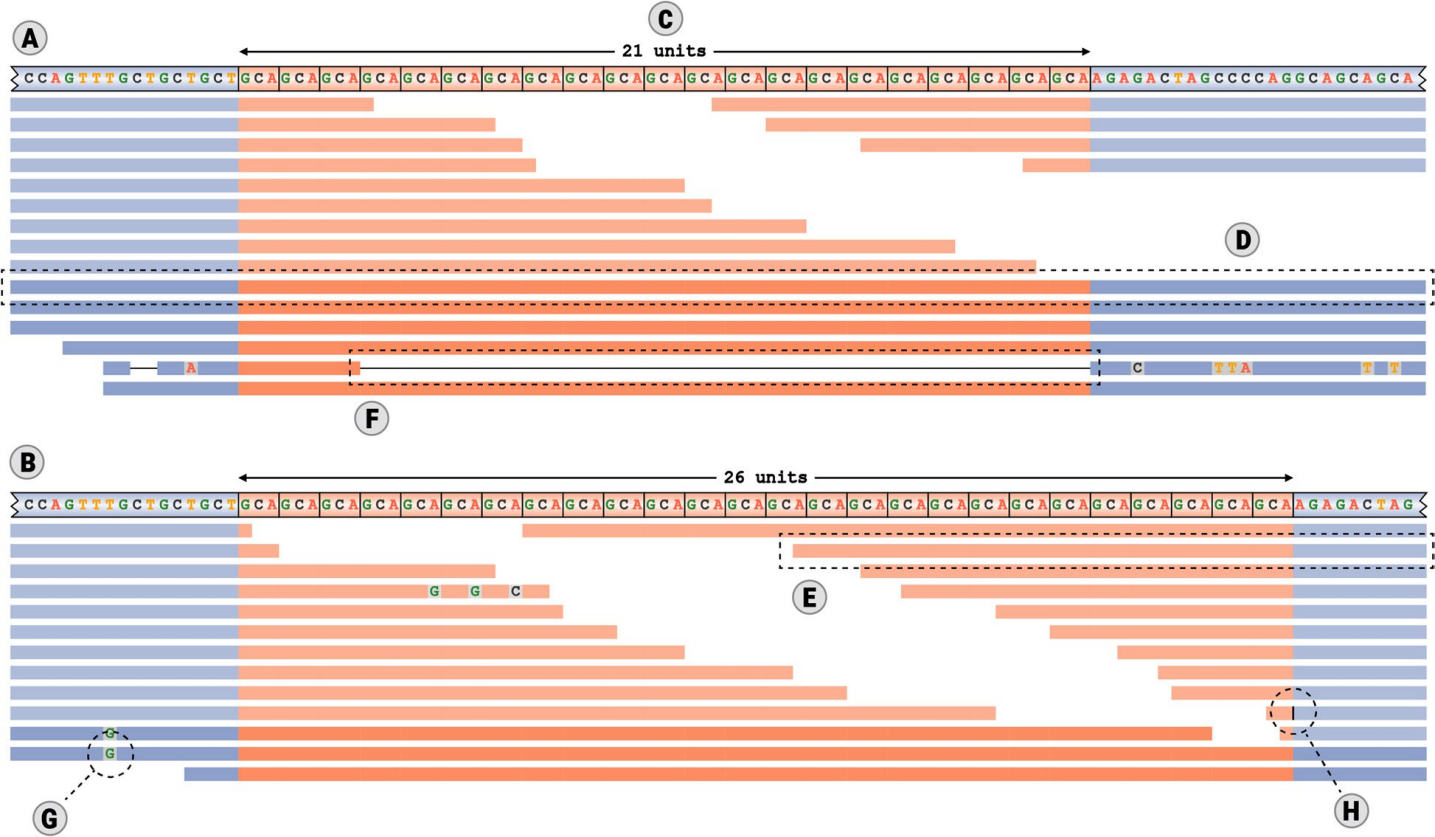
An example plot generated by REViewer: **A**, **B** local haplotype sequences with read alignments; **C** estimated STR allele length; **D** a read that fully spans the STR sequence; **E** a flanking read that partially overlaps the STR; this read is depicted in a fainter color because it can be assigned to either haplotype; **F** a deletion in the read alignment; **G** a single-base mismatch; and **H** an insertion site

Here we present the Repeat Expansion Viewer (REViewer), a novel method for visualizing short read sequencing data in genomic regions containing one or multiple STRs (Fig. 1). REViewer has been designed to work with the read alignments produced by ExpansionHunter [8, 9], though it will work with any repeat genotyping software that produces output in the appropriate format. We also describe FlipBook, a companion image viewer that is designed for manual curation of large collections of images generated by REViewer.

## Implementation

### Overview

**Fig. 2:**
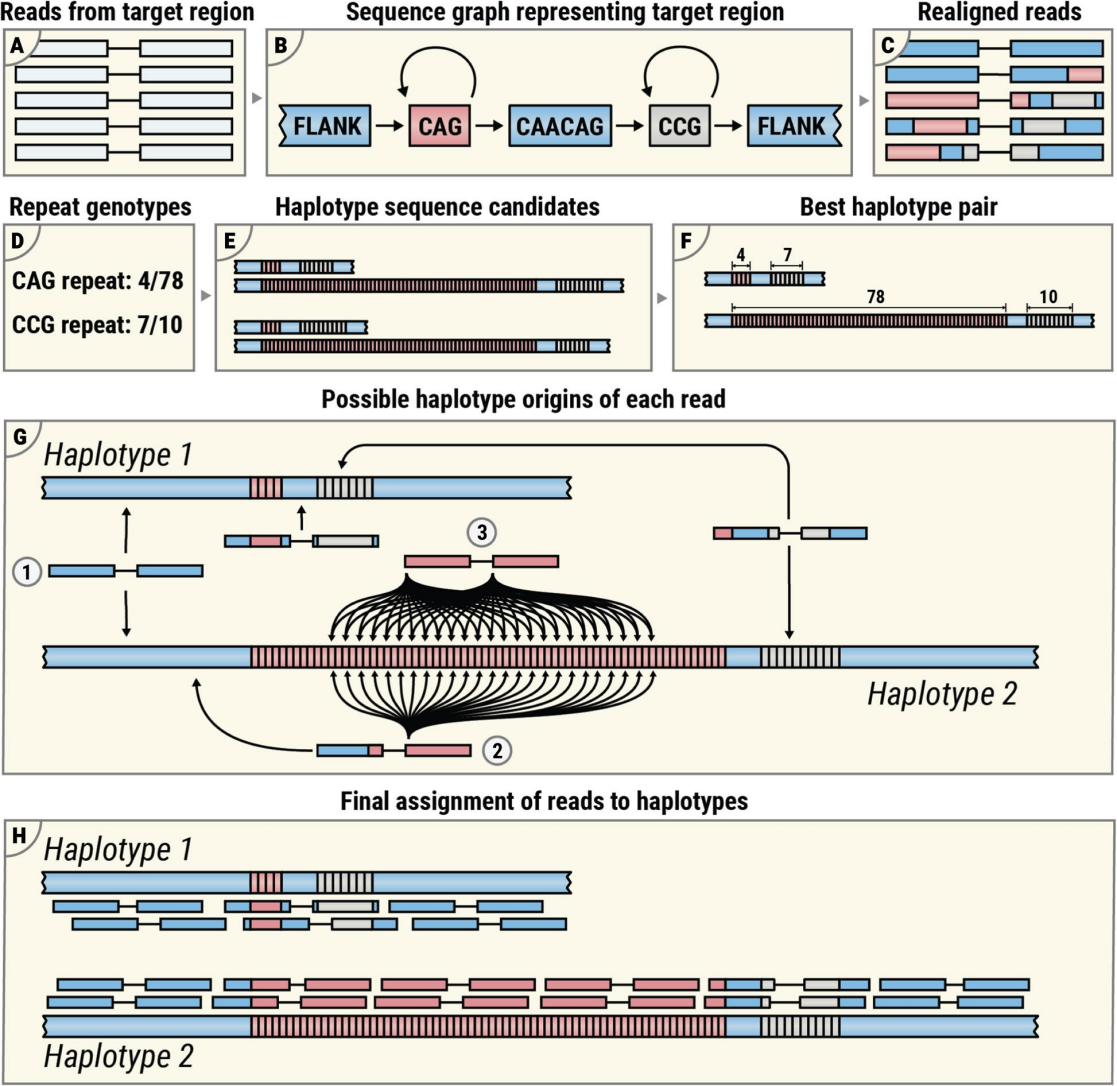
An overview of the pileup generation algorithm: **A**–**C** reads originating in the region containing target STRs are realigned using the sequence graph aligner within ExpansionHunter software; **D**, **E** putative pairs of haplotype sequences are generated from repeat genotypes; **F** a haplotype pair that has the highest consistency with read alignments is selected; **G** possible alignments of each read to each haplotype sequence are generated from the original sequence-graph alignments; and **H** pairs of read alignments that correspond to the most consistent fragment length are selected for each read pair and then one of these is randomly selected for visualization

REViewer is designed to work with the BAM [10] and VCF files [11] generated by ExpansionHunter [8, 9], a commonly used method for repeat genotyping. The VCF file is used to obtain repeat genotypes while the BAM file contains reads realigned to a sequence graph representing the entire repeat region (Fig. 2A–D). Additionally, we created a wrapper script that accepts regular BAM files containing alignments of reads to a linear genome and a tab-separated file containing reference coordinates of the target STRs, repeat units, and repeat genotypes making it possible to use REViewer with other software (Additional file 1: Supplementary methods).

### Read pileup generation

**Fig. 3:**
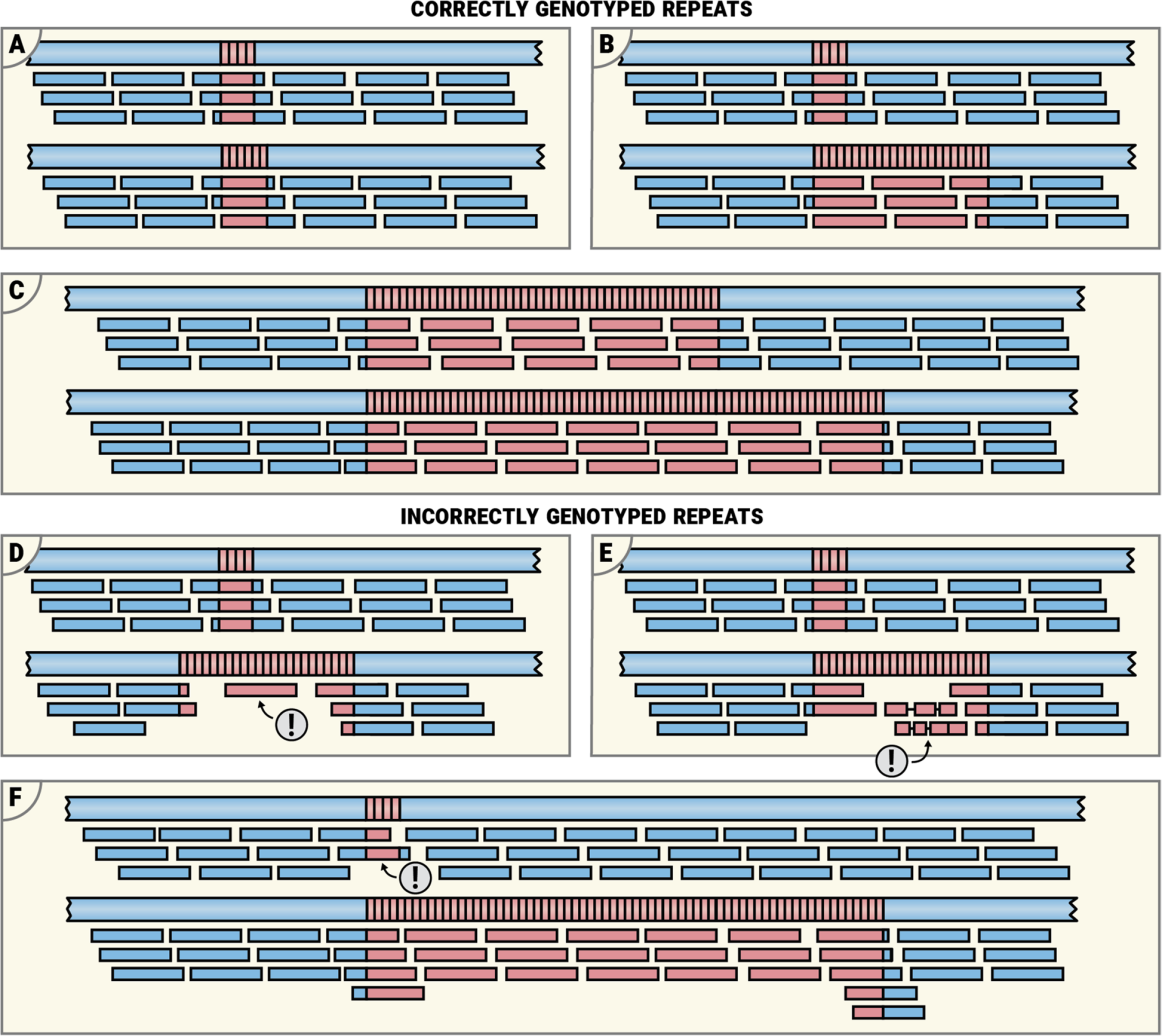
Examples of read pileups. Pileups corresponding to correctly genotyped repeats: **A** both repeat alleles are short; **B** one allele is expanded; and **C** both alleles are expanded. Pileups corresponding to incorrectly genotyped repeats (problem areas are marked with an exclamation sign): **D** expanded allele is supported by just one read suggesting that its size is overestimated; **E** expanded allele is supported by poorly aligning reads (each containing multiple indels) suggesting that the reads are incorrectly mapped and that size of the repeat is overestimated; **F** the short allele is supported by just one spanning read suggesting that this allele is not real and that both alleles are expanded

Read pileups are generated using genotypes of all STRs present at the target region and reads aligned to a sequence graph representing the region (Fig. 2A–D; [9]). For repeats on diploid chromosomes, REViewer constructs all possible pairs of haplotype sequences from the STR genotypes. For example, if a region contains two STRs then there are four possible haplotypes that can be formed and two possible haplotype pairings (Fig. 2E). The reads are next aligned to all haplotype pairs by transforming the graph alignments from the BAM file generated by ExpansionHunter into linear alignments. The haplotype pair that yields the highest cumulative read alignment score is selected for visualization (Fig. 2F). Loci with a single STR or on haploid chromosomes have unambiguous haplotypes and so the haplotype sequence selection steps are skipped. Next, for each read pair, REViewer finds the top-scoring alignments to any haplotype sequence (Fig. 2G). A read pair originating completely within a sequence surrounding the repeats and shared by all haplotypes has exactly one alignment position on each haplotype (Fig. 2G, 1). When one mate originates fully within the repeat, the number of positions for the read pair increases linearly with the repeat length (Fig. 2G, 2). In contrast, when both mates originate inside the repeat, the number of positions increases quadratically (Fig. 2G, 3). For read pairs where one or both mates have multiple alignments, REViewer selects pairs of alignments that correspond to fragment length closest to the mean fragment length calculated for read pairs mapping to the flanking regions surrounding the repeats. Finally, REViewer generates read pileup by selecting one pair of alignments at random for each read pair (Fig. 2H).

This algorithm is based on the idea that if a given locus is sequenced well and each constituent repeat is genotyped correctly, then it is possible to distribute the reads to achieve an even coverage of each haplotype. Importantly, assignment of some reads to the correct haplotype of origin will be ambiguous, especially in cases when the repeats are homozygous, and the resulting haplotypes are identical.

Pileups corresponding to correctly genotyped repeats are characterized by a relatively even read coverage of both alleles (Fig. 3A–C). At the typical whole-genome sequencing depths (30–60×), each position of a haplotype sequence is expected to be covered by many reads (15–30), though the coverage may dip in certain regions due to technical factors like GC bias. For repeats much shorter than the read length, this implies the presence of multiple spanning reads (Fig. 3, both alleles on panel A and short allele on panel B). Repeats much larger than the read length are expected to contain multiple in-repeat reads (Fig. 3, long allele on panel A and both alleles on panel B). An incorrectly called expanded allele might have low sequencing depth inside the repeat compared to the depth of the region surrounding the repeat (Fig. 3, long allele on panel D). Additionally, the presence of multiple indels in the alignments of in-repeat reads indicates that the reads may not be correctly aligned (possibly due to sequencing errors) and that the size of the repeat may be overestimated (Fig. 3E). Finally, a short allele supported by one or very few spanning reads may not be real. For instance, the short allele depicted on panel F of Fig. 3 is supported by just one spanning and one flanking read, which is less than expected based on the coverage of the surrounding region. There is also a slight excess of the flanking reads on the long allele of this repeat. Taken together, these observations suggest that (a) the single spanning read may be a result of an incorrect alignment and (b) the correct genotype is likely to be a double expansion. Some real examples corresponding to the scenarios depicted in Fig. 3 are included in online documentation [12].

### FlipBook image viewer

In many situations, researchers may wish to look at STR genotypes for a variety of known repeat loci across many samples. To simplify the painstaking manual task of reviewing many REViewer pileups and recording the results of manual review, we developed FlipBook—a photo-album-like application that lets a user quickly assess pileups on their local hard drive and record notes about each one. Additional features of this software include (1) displaying custom information above the images—such as affected status and STR locus information; (2) customizing the questions a user can answer about each image; and (3) displaying more than one image at a time—such as when evaluating data from multiple family members.

## Results

### A concordance study

To solicit feedback on REViewer and FlipBook and create training materials for new REViewer users, we performed a concordance study involving 12 scientists (analysts). We used a collection of whole-genome sequencing (WGS) samples described in a recent study of subjects with suspected neurological disorders [13] and additional samples with PCR-validated *FMR1* and *DMPK* repeats from the 100,000 Genomes Project (Additional file 1: Supplementary methods; Additional file 2: Table S1). The *HTT*, *TBP*, *AR*, *ATXN3*, *ATN1*, *ATXN2*, *ATXN7*, *ATXN1*, *CACNA1A*, *DMPK*, *PPP2R2B*, *FXN*, *FMR1*, and *C9orf72* STR loci were genotyped in these samples with ExpansionHunter (EH) and also tested with PCR. To emulate a practical assessment strategy, only the STRs for which the size confidence interval reported by EH overlapped or exceeded an intermediate or full expansion threshold were selected for review. This totaled 133 STR genotypes (one genotype per sample) across all 14 STR loci. REViewer read pileups corresponding to these 133 genotypes (Additional file 2: Table S1) were evaluated by the analysts using FlipBook software. The analysts categorized the genotyped STRs into normal, intermediate expansion, full expansion, and biallelic expansion categories. The verdicts were recorded by FlipBook for subsequent analysis.

**Fig. 4:**
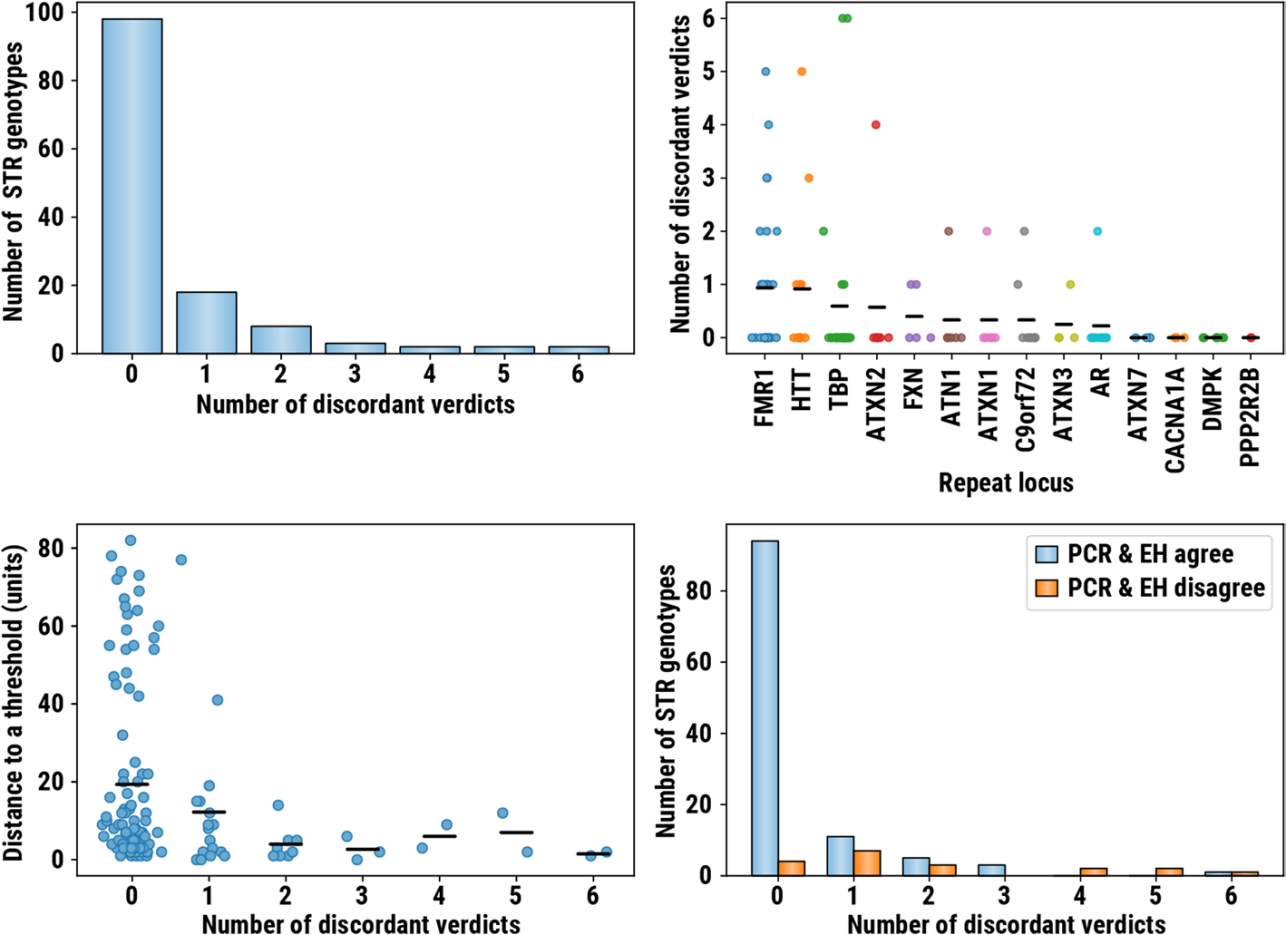
**A** Counts of STR genotypes where the specified number of analysts disagreed with the consensus verdict (discordant verdict); **B** distribution of discordant verdict counts stratified by STR locus; **C** distribution of distances between STR sizes estimated by ExpansionHunter (EH) and pathogenic threshold; and **D** the counts of repeat genotypes where NGS and PCR agree and disagree

To measure consistency of analysts’ responses, we calculated the number of discordant verdicts for each genotyped STR. A verdict was defined as discordant if it differed from the most common consensus verdict. The majority of verdicts were highly consistent—three or more analysts disagreed with the consensus verdict for only 9 out of 133 genotyped STRs (Fig. 4A). The mean number of STRs with discordant verdicts was below one for all STR loci (Fig. 4B). *FMR1* repeats had the largest number of discordant verdicts (0.94 on average) which is consistent with earlier observations that the *FMR1* locus is harder to size accurately as the repeat becomes long [8]. Disagreements in verdicts arose for STRs where the size estimate was close to the pathogenic threshold (Fig. 4C).

Next, we compared the verdicts for repeats where EH and PCR-based calls agreed to those where they disagreed (using binary categorization for *FMR1* and *C9orf72* repeats; see below). When EH and PCR-based calls agreed, most repeats (94 out of 114) had no discordant verdicts but when the EH and PCR-based calls disagreed, only a few (4 out of 19) had no discordant verdicts (Fig. 4D). This suggests that, with additional training, the information presented in REViewer/FlipBook visualizations can be used to reduce the false-positive rate for many known pathogenic loci. To provide such training, we created online documentation that consists of both a tutorial describing how to review the pileups (Fig. 3 and [14]) and a repository of pileups corresponding to harder to interpret correct and incorrect calls. On average, PCR concordance after manual review was similar to the raw ExpansionHunter genotype calls (Additional file 1: Supplementary methods). Unsurprisingly, the three highest-performing analysts had substantial prior experience with evaluating STR calls and used more subtle image features to achieve higher-than-average concordance.

### *FMR1* and *C9orf72* repeat loci

Due to the difficulty of distinguishing between the intermediate and full expansions of *FMR1* [8] and *C9orf72* repeats (full expansions start at 600bp and 360bp respectively), they were categorized into two categories: normal and expanded. This categorization also reflects the fact that, in practice, the ability to distinguish between normal and abnormal sized repeats is more important than being able to accurately classify intermediate versus expanded alleles. Individuals identified with abnormal-sized repeats that may explain their phenotype or place them at risk for disease or passing on an expandable repeat are likely to be sent for orthogonal confirmation testing, regardless of whether the estimated STR size is in the intermediate or pathogenic range.

### Annotating interruptions with REViewer

REViewer visualizations also display deviations from the predicted sequence and this can allow users to identify STR interruptions. To demonstrate this functionality, we assessed the pileups of 29 *FMR1* reference samples [8] with prior TP-PCR data [15, 16] on repeat length and number and position of AGG interruptions. The concordance between AGG-interruption maps derived from the REViewer pileups and TP-PCR was evaluated. Additional file 6  shows the read pileups and TP-PCR electropherograms of two representative samples–a normal male (NA06890, panel A) and an intermediate female (NA20234, panel B). NA06890 with 30 repeat units has two AGGs evident in the pileups as mismatches at repeat positions 11 and 21. This (CGG)_10_AGG(CGG)_9_AGG(CGG)_9_ structure is consistent with TP-PCR. In NA20234, the pileups show the clear assignment of reads to the correct haplotypes, a 31-repeat normal and a 46-repeat intermediate allele with (CGG)_10_AGG(CGG)_9_AGG(CGG)_10_ and (CGG)_9_AGG(CGG)_9_AGG(CGG)_13_AGG(CGG)_12_ structures, respectively. The TP-PCR analysis had consistent repeat structures, but the superimposing amplicon peaks from the two *FMR1* alleles in some heterozygous female samples with complex repeat structures may make AGG-interruption mapping relatively harder with TP-PCR [15].

Of the 44 alleles assessed in total (14 males and 15 females), the AGG-interruption maps of 38 alleles derived from the pileups were consistent with that of TP-PCR (Additional file 1: Supplementary methods). Concordant results (86.36%) were noted for 20/23 normal, 5/5 intermediate, 6/8 premutation, and 7/8 3′-uninterrupted full-mutation alleles.

Among the six discrepant alleles, the normal alleles of NA20243 and NA20240 had an incorrect ExpansionHunter genotype and inadequate spanning/flanking reads in the pileups that hampered the interpretation of AGG interruptions. The normal allele of NA20244 was sized one CGG-repeat less by ExpansionHunter, and the pileup and TP-PCR structures were (CGG)_9_AGG(CGG)_8_AGG(CGG)_21_ and (CGG)_9_AGG(CGG)_9_AGG(CGG)_21_, respectively. We could not resolve the AGG-interruption pattern of the premutation allele in NA20240 due to the ambiguity in the assignment of reads to the two haplotypes as ExpansionHunter genotyped this heterozygous premutation sample (30/80 repeats) as homozygous premutation (95/95 repeats). In NA06907, the premutation haplotype did not have sufficient reads to support the TP-PCR’s (CGG)_10_AGG(CGG)_80_ repeat structure. In NA07537, we could not confidently ascertain the interruption pattern of the full-mutation allele from the pileups because of the ambiguity in read assignment. In general, the TP-PCR data supported the presence of uninterrupted CGG-repeats at the 3′-ends of the full-mutation alleles. Nonetheless, in two full-mutation males (NA06852 and NA06897), the pileup visualization enabled the detection of an AGG interruption at the 5′-end of the full-mutation, which, as expected, was not evident from the TP-PCR analyses that target the 3′-ends. See Additional file 3 for pileups and TP-PCR profiles of additional *FMR1* intermediate, premutation, and full-mutation samples.

### Comparison with haplotype-resolved assemblies

To further explore possible uses of REViewer, we extracted genotypes of 36 STRs from a recent long-read assembly of NA12878 genome [17, 18]. All but two genotypes were either identical or disagreed by one repeat unit. In the two remaining cases, ExpansionHunter reported a heterozygous instead of homozygous genotype with many high-quality spanning reads as evidence. Notably, the local haplotypes determined by REViewer for the *CNBP* locus agreed with the long-read assembly. This locus is arguably the most complex locus assessed here because it contains three adjacent STRs (Additional file 4: Fig S1).

## Discussion

REViewer enables visualization of sequencing data in genomic regions containing one or more tandem repeats by reconstructing local haplotypes containing the repeats of interest and then generating read pileups over these haplotypes. FlipBook, the companion image viewer for REViewer, enables interactive curation of large sets of read pileups and subsequent output of the curation results into a file. We have shown that REViewer and FlipBook can be used for a wide range of purposes including quality assessment of repeat expansion calls produced by bioinformatics pipelines and studies of interruptions and other imperfections in repeat sequences. Additionally, these visualizations are a valuable tool for continued development of new methods for STR analysis.

To create a user guide for REViewer, we performed a concordance study involving 12 scientists involved in STR research. This study highlights a range of pileup features (Fig. 3 and [14]) that can help to identify lower confidence calls and potential genotyping errors. This information, together with representative example pileups, was documented in the online user guide. The concordance study also helped to highlight some important limitations of REViewer. Namely, pileups cannot be used to determine if the size of a long repeat expansion is underestimated. This is because pileups of longer repeats missing in-repeat reads can be indistinguishable from pileups corresponding to shorter repeats.

REViewer visualization offers the unique advantage of analyzing interruptions at both the 5′- and 3′-ends of the repeat sequences and determining the exact sequences of the interrupting motifs. In the extremely GC-rich *FMR1* repeat locus, which is prone to coverage bias, REViewer achieved an overall 86.36% concordance across normal, intermediate, premutation, and full-mutation genotypes. Interruptions are observed in a number of repeat expansions and their presence or absence may modify the pathogenicity, disease severity or presentation [19–21]. The ability to visualize and assess interruptions is a valuable addition to bioinformatics repeat expansion pipelines. It would be difficult to piece together this information by inspecting alignments of reads to a reference genome using the general-purpose visualization tools like the Integrative Genomics Viewer [2] and JBrowse [3] (Additional file 5: Fig S2). We believe that future improvements to ExpansionHunter genotyping and REViewer’s ability to consider interruptions during the assignment of reads to the haplotypes will enable even better annotations of STR interruptions.

We are planning to continue improving REViewer and FlipBook in response to feedback from the user community. In particular, we are considering extending REViewer to support other variant types.

## Conclusions

Clinical applications of sequencing data continue to rapidly expand. Bioinformatics pipelines for genome analysis continue to increase the types of variants that they profile and incorporate even more difficult regions of the genome. Visualization of sequencing evidence supporting more complex variants requires specialized visualization algorithms and user interfaces. The work here demonstrates that variant-specific visualizations that augment general purpose visualization tools are a pragmatic strategy to increase the utility of bioinformatics pipelines. REViewer and FlipBook are available under open-source licenses at https://github.com/illumina/REViewer and https://github.com/broadinstitute/flipbook respectively.

## Availability and requirements

Project name: REViewer and FlipBook

Project home page: https://github.com/Illumina/REViewer, https://github.com/broadinstitute/flipbook/

Operating systems: REViewer: Linux and macOS; FlipBook: Linux, macOS, and Windows

Programming languages: C++ (REViewer) and Python (FlipBook)

License: GNU GPLv3 (REViewer) and MIT (FlipBook)

## Supplementary Information

**Additional file 1: Supplementary methods.** Description of the concordance study dataset; Description of the wrapper script; Evaluation of manual review performance; Comparison with haplotype-resolved assemblies, Comparison with other visualization software; REViewer allele structure of *FMR1* reference samples; Comparison of STR genotypes extracted from long-read genome assembly.

**Additional file 2: Table S1.** A description of 133 repeats used for the concordance study. Each row describes the basic sample/repeat information (columns Sample, Sex, Locus), repeat size estimated by PCR and EH (columns PCR_short, PCR_long, EH_short, EH_long, EH_short_CI, EH_long_CI), size thresholds (columns Premuation, Pathogenic), verdicts based on PCR & EH size estimates (columns PCR_verdict, EH_verdict), and analyst verdicts (columns Analyst 1, …, Analyst 12).

**Additional file 3. **A slide deck with REViewer pileups and TP-PCR profiles of additional *FMR1* intermediate, premutation, and full-mutation samples (1) NA20232, (2) NA20230, (3) CD00014, (4) GM06892, (5) GM06852, (6) GM07063.

**Additional file 4: Figure S1.** REViewer pileup plots for some NA12878 STRs: (A) *ATXN10*, (B) *RFC1*, (C) *CNPB*.

**Additional file 5: Figure S2.** Read pileups in a region surrounding DMPK repeat expansion generated by (A) JBrowse, (B) IGV, and (C) REViewer.

**Additional file 6: Figure S3.** REViewer read pileups and TP-PCR electropherograms of FMR1 repeats in samples (A) NA06890 and (B) NA20234

## Data Availability

• REViewer’s source code and binaries: https://github.com/Illumina/REViewer [14] • FlipBook’s source code: https://github.com/broadinstitute/flipbook [22] • Pileups for 133 STR genotypes: https://github.com/broadinstitute/StrPileups [23]

## Abbreviations

EH: ExpansionHunter
In-repeat read: Read fully contained in the repeat sequence
REViewer: Repeat expansion viewer
STR: Short tandem repeat
TP-PCR: Triplet primed PCR
