# Supplementary material for: REViewer: haplotype-resolved visualization of read alignments in and around tandem repeats
Source: Genome Med. 2022 Aug 11;14:84. doi: 10.1186/s13073-022-01085-z (PMC9367089; doi:10.1186/s13073-022-01085-z)
Supplement: Supplementary file 1 — Additional file 1: Supplementary methods. Description of the concordance study dataset; Description of the wrapper script; Evaluation of manual review performance; Comparison with haplotype-resolved assemblies, Comparison with other visualization software; REViewer allele structure of FMR1 reference samples; Comparison of STR genotypes extracted from long-read genome assembly. [file 13073_2022_1085_MOESM1_ESM.docx]

# Supplementary methods

## Description of the concordance study dataset

Whole genome sequencing for 868 STRs corresponding to 398 subjects were included in the dataset. A total of 793 out of 868 cases are described in [(1)](https://paperpile.com/c/ifjewP/TMR8F), and an additional 75 PCR-validated *FMR1* and *DMPK* repeats came from 100,000 Genomes Project samples. The samples were de-identified and do not correspond to the same IDs as in [(1)](https://paperpile.com/c/ifjewP/TMR8F). Only the 133 STRs whose size confidence interval overlapped or exceeded an intermediate or full expansion threshold were selected for review (**Additional file 2**: Table S1).

## Description of the wrapper script

The wrapper script works by creating read pileups for loci of interest in one or many BAM files. As an input, it requires either a folder or comma separated list of aligned and indexed BAM files as well as a regions (BED) file with coordinates of an STR locus and the repeated motif. The script requires access to the reference genome and REViewer, ExpansionHunter, and SAMtools [(2)](https://paperpile.com/c/ifjewP/YCLc8) executables which will be used to genotype specified STR loci and create read pileups. Additionally, a custom genotype (such as one determined by other methods) can be specified in the BED file to override ExpansionHunter's estimated genotype. REViewer will then use and adjust read visualizations for this custom genotype. REViewer will then use and adjust read visualizations for this custom genotype.

While running the script, a BED file will be converted into a temporary variant catalog where each region becomes a record in the catalog and which will be used to create read pileups for input file(s). Visualizations in SVG format will be saved into the specified output folder with the option to create a HTML file that contains all results for the run for easy reviewing.

## Evaluation of manual review performance

We evaluated analyst responses to determine the degree to which manual review improved concordance with PCR. We defined concordance as agreement on whether the number of repeats in a sample is normal or is above the normal threshold. The concordance with PCR after manual review was 121.75 out of 133 samples on average, while raw ExpansionHunter genotypes were concordant with PCR for 121 out of 133 samples. The three highest-performing analysts had PCR concordance of 124 or 125 out of 133 samples. Unsurprisingly, all three had substantial prior experience with evaluating STR calls and REViewer images as part of their research.

We then looked at REViewer images where the highest-performing analysts responded differently from other analysts. There we identified the subtle image features these analysts used to achieve the higher-than-average concordance.

To flag genotypes that were overestimated by ExpansionHunter, the highest-performing analysts identified cases where:
- an above-normal genotype significantly relied on evidence from a single read (examples: [GE_case253](https://broadinstitute.github.io/StrPileups/page_GE_case253.html), [GE_case630](https://broadinstitute.github.io/StrPileups/page_GE_case630.html), [GE_case755](https://broadinstitute.github.io/StrPileups/page_GE_case755.html), [GE_case852](https://broadinstitute.github.io/StrPileups/page_GE_case852.html))
- In-repeat reads (IRRs) contained many insertions or deletions (examples: [GE_case620](https://broadinstitute.github.io/StrPileups/page_GE_case620.html), [GE_case623](https://broadinstitute.github.io/StrPileups/page_GE_case623.html))

To flag genotypes that were underestimated by ExpansionHunter, the analysts noticed:

- insertion symbols in multiple spanning reads ([GE_case859](https://broadinstitute.github.io/StrPileups/page_GE_case859.html), [GE_case25](https://broadinstitute.github.io/StrPileups/page_GE_case80.html))
- soft-clipped regions of spanning reads (examples: [GE_case80](https://broadinstitute.github.io/StrPileups/page_GE_case80.html))
- matching interruptions in left-flanking reads with interruptions in right-flanking reads

## Comparison with haplotype-resolved assemblies

To further benchmark ExpansionHunter genotype calls, we extracted genotypes of 36 STRs from a recent long-read assembly of NA12878 genome [(3,4)](https://paperpile.com/c/ifjewP/n3O47+TZWQf). The genotypes of 30 STRs were identical between the two genotyping methods (see table below). The genotypes of AR, ATXN8OS, FMR1, and TBP repeats disagreed by only a single motif copy. Only ExpansionHunter detected an allele of 65bp for ATXN10 STR and an allele of size 60bp for RFC1 STR. These alleles were supported by many high-quality spanning reads (**Additional file 4**: Fig S1). Notably, the local haplotypes determined by REViewer for CNBP locus agreed with the long-read assembly. This locus is arguably the most complex locus assessed here because it contains three adjacent STRs (**Additional file 4**: Fig S1).

## Comparison with other visualization software

To further highlight the usefulness of REViewer compared to general-purpose visualization software, we generated read pileups in a region surrounding the *DMPK* repeat expansion using JBrowse, IGV, and REViewer (**Additional file 5**: Fig S2). As expected, IGV and JBrowse do not display reads fully contained in the expanded repeat and therefore are difficult to use for assessment of repeat expansion calls.

## REViewer allele structure of *FMR1* reference samples

| Coriell ID | Coriell allele size (genotype) | EH allele size | REViewer allele structure |
| --- | --- | --- | --- |
| NA07175 | 23/30 (NL) | 23/30 | (CGG)_13_AGG(CGG)_9_/(CGG)_10_AGG(CGG)_9_AGG(CGG)_9_ |
| NA06890 | 30 (NL) | 30 | (CGG)_10_AGG(CGG)_9_AGG(CGG)_9_ |
| NA07174 | 30 (NL) | 30 | (CGG)_10_AGG(CGG)_9_AGG(CGG)_9_ |
| NA07538 | 29/29 (NL) | 29/29 | (CGG)_9_AGG(CGG)_9_AGG(CGG)_9_/(CGG)_9_AGG(CGG)_9_AGG(CGG)_9_ |
| NA07541 | 29/31 (NL) | 29/31 | (CGG)_9_AGG(CGG)_9_AGG(CGG)_9_/(CGG)_10_AGG(CGG)_10_AGG(CGG)_9_ |
| NA20243 | 29/41 (NL) | 29/32 | (CGG)_9_AGG(CGG)_9_AGG(CGG)_9_/could not be ascertained |
| NA20238 | 29/30 (NL) | 29/30 | (CGG)_9_AGG(CGG)_9_AGG(CGG)_9_/(CGG)_9_AGG(CGG)_9_AGG(CGG)_10_ |
| NA20244 | 41 (NL) | 40 | (CGG)_9_AGG(CGG)_8_AGG(CGG)_21_ |
| NA20234 | 31/46 (IM) | 31/46 | (CGG)_10_AGG(CGG)_9_AGG(CGG)_10_/  (CGG)_9_AGG(CGG)_9_AGG(CGG)_13_AGG(CGG)_12_ |
| NA20232 | 46 (IM) | 45 | (CGG)_9_AGG(CGG)_35_ |
| NA20235 | 29/45 (IM) | 29/40 | (CGG)_9_AGG(CGG)_9_AGG(CGG)_9_/(CGG)_10_AGG(CGG)_29_ |
| NA20236 | 31/53 (IM) | 31/44 | (CGG)_10_AGG(CGG)_9_AGG(CGG)_10_/(CGG)_44_ |
| NA20230 | 53 (IM) | 65 | (CGG)_65_ |
| CD00014 | 56 (PM) | 58 | (CGG)_9_AGG(CGG)_9_AGG(CGG)_38_ |
| NA20231 | 76 (PM) | 82 | (CGG)_10_AGG(CGG)_71_ |
| NA20242 | 30/73 (PM) | 30/67 | (CGG)_10_AGG(CGG)_9_AGG(CGG)_9_/(CGG)_9_AGG(CGG)_9_AGG(CGG)_47_ |
| NA06892 | 93 (PM) | 73 | (CGG)_10_AGG(CGG)_62_ |
| NA20240 | 30/80 (PM) | 95/95 | could not be ascertained/could not be ascertained |
| NA06907 | 29/85 (PM) | 29/95 | (CGG)_9_AGG(CGG)_9_AGG(CGG)_9_/could not be ascertained |
| NA06896 | 23/95-120-140 (PM) | 23/80 | (CGG)_13_AGG(CGG)_9_/(CGG)_10_AGG(CGG)_69_ |
| NA06891 | 118 (PM) | 110 | (CGG)_110_ |
| NA07862 | 501-550 (FM) | 99 | (CGG)_99_ |
| NA07294 | n.a (FM) | 102 | (CGG)_102_ |
| NA04025 | 645 (FM) | 112 | (CGG)_111_ |
| NA20239 | 20/183-193 (FM) | 20/103 | (CGG)_10_AGG(CGG)_9_/(CGG)_103_ |
| NA07063 | n.a (FM) | 32/93 | (CGG)_9_AGG(CGG)_22_/(CGG)_93_ |
| NA06852 | >200 (FM) | 81 | (CGG)_10_AGG(CGG)_70_ |
| NA06897 | 477 (FM) | 80 | (CGG)_10_AGG(CGG)_69_ |
| NA07537 | 28-29/>200 (FM) | 29/72 | (CGG)_9_AGG(CGG)_9_AGG(CGG)_9_/could not be ascertained |

NL, normal; IM, intermediate; PM, premutation; FM, full-mutation; n.a, not available; EH, ExpansionHunter

Allele size includes AGG interruptions

## Comparison of NA12878 STR genotypes extracted from long-read genome assembly [(3)](https://paperpile.com/c/ifjewP/n3O47) and estimated by ExpansionHunter

| Locus | Motif | Assembly | ExpansionHunter |
| --- | --- | --- | --- |
| AFF2 | GCC | 60/60 | 60/60 |
| AR | GCA | 66/75 | 63/75 |
| ATN1 | CAG | 57/57 | 57/57 |
| ATXN1 | TGC | 90/93 | 90/93 |
| ATXN10 | ATTCT | 80/80 | 65/80 |
| ATXN2 | GCT | 66/69 | 66/69 |
| ATXN3 | GCT | 60/63 | 60/63 |
| ATXN7 | GCA | 30/36 | 30/36 |
| ATXN7 | GCC | 12/12 | 12/12 |
| ATXN8OS | CTA | 48/48 | 45/48 |
| ATXN8OS | CTG | 39/39 | 39/39 |
| C9ORF72 | GGCCCC | 12/30 | 12/30 |
| CACNA1A | CTG | 33/36 | 33/36 |
| CBL | CGG | 33/33 | 33/33 |
| CNBP | CAGG | 60/60 | 60/60 |
| CNBP | CAGA | 42/54 | 42/54 |
| CNBP | CA | 32/36 | 32/36 |
| DIP2B | GGC | 21/48 | 21/48 |
| DMPK | CAG | 15/39 | 15/39 |
| FMR1 | CGG | 93/93 | 90/93 |
| FXN | A | 27/27 | 27/27 |
| FXN | GAA | 27/27 | 27/27 |
| GIPC1 | CCG | 33/33 | 33/33 |
| GLS | GCA | 24/42 | 24/42 |
| HTT | CAG | 48/54 | 48/54 |
| HTT | CCG | 27/36 | 27/36 |
| JPH3 | CTG | 42/48 | 42/48 |
| NIPA1 | GCG | 24/24 | 24/24 |
| NOP56 | GGCCTG | 24/24 | 24/24 |
| NOP56 | CGCCTG | 12/18 | 12/18 |
| PABPN1 | GCG | 18/18 | 18/18 |
| PHOX2B | GCN | 60/60 | 60/60 |
| PPP2R2B | GCT | 30/42 | 30/42 |
| RFC1 | AARRG | 40/40 | 40/60 |
| TBP | GCA | 111/111 | 111/114 |
| TCF4 | CAG | 51/81 | 51/81 |

## References

1. [Ibañez K, Polke J, Hagelstrom RT, Dolzhenko E, Pasko D, Thomas ERA, et al. Whole genome sequencing for the diagnosis of neurological repeat expansion disorders in the UK: a retrospective diagnostic accuracy and prospective clinical validation study. Lancet Neurol [Internet]. 2022 Mar [cited 2022 Apr 17];21(3). Available from:](http://paperpile.com/b/ifjewP/TMR8F) <https://pubmed.ncbi.nlm.nih.gov/35182509/>

2. [Danecek P, Bonfield JK, Liddle J, Marshall J, Ohan V, Pollard MO, et al. Twelve years of SAMtools and BCFtools. Gigascience [Internet]. 2021 Feb 16;10(2). Available from:](http://paperpile.com/b/ifjewP/YCLc8) <http://dx.doi.org/10.1093/gigascience/giab008>

3. [Ebert P, Audano PA, Zhu Q, Rodriguez-Martin B, Porubsky D, Bonder MJ, et al. Haplotype-resolved diverse human genomes and integrated analysis of structural variation. Science [Internet]. 2021 Apr 2;372(6537). Available from:](http://paperpile.com/b/ifjewP/n3O47) <http://dx.doi.org/10.1126/science.abf7117>

4. [Zook JM, McDaniel J, Olson ND, Wagner J, Parikh H, Heaton H, et al. An open resource for accurately benchmarking small variant and reference calls. Nat Biotechnol. 2019 May;37(5):561–6.](http://paperpile.com/b/ifjewP/TZWQf)
