# Supplementary material for: REViewer: haplotype-resolved visualization of read alignments in and around tandem repeats
Source: Genome Med. 2022 Aug 11;14:84. doi: 10.1186/s13073-022-01085-z (PMC9367089; doi:10.1186/s13073-022-01085-z)
Supplement: Supplementary file 3 — Additional file 3. A slide deck with REViewer pileups and TP-PCR profiles of additional FMR1 intermediate, premutation, and full-mutation samples (1) NA20232, (2) NA20230, (3) CD00014, (4) GM06892, (5) GM06852, (6) GM07063. [file 13073_2022_1085_MOESM3_ESM.pptx]

## Slide 1
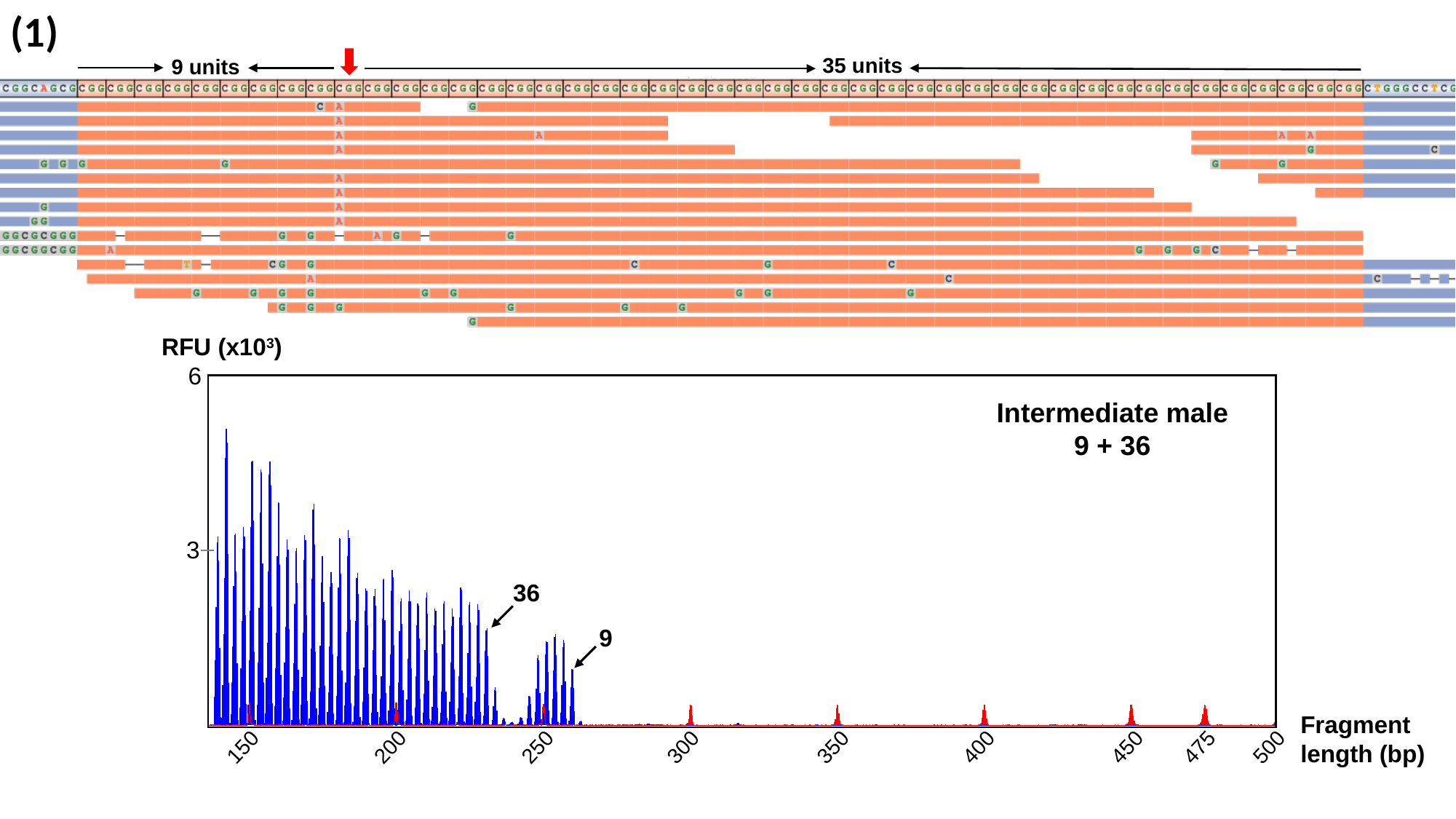

(1)
35 units
9 units
RFU (x103)
6
Intermediate male
9 + 36
3
36
9
Fragment length (bp)
150
200
250
300
350
400
450
475
500

## Slide 2
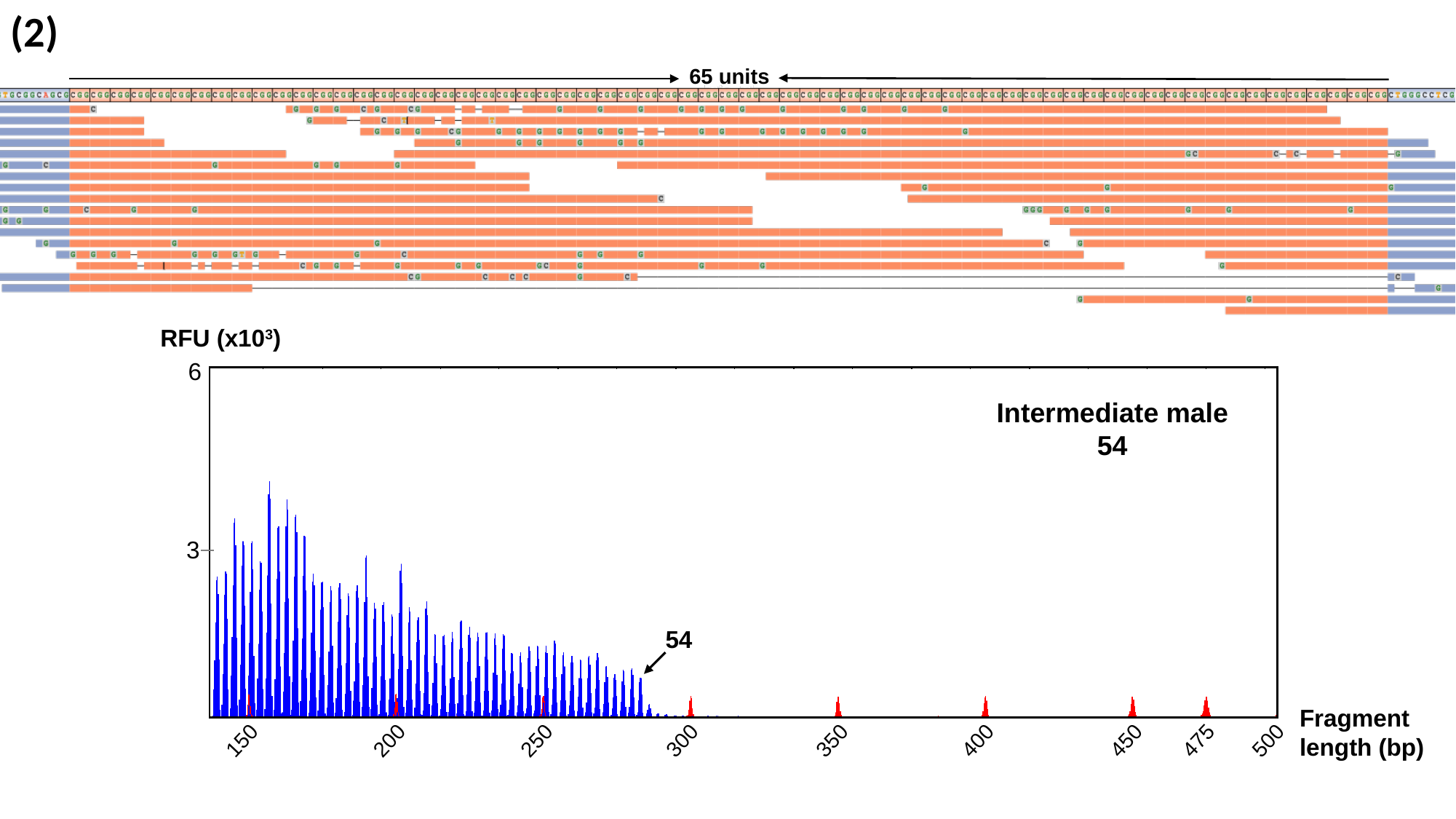

(2)
65 units
RFU (x103)
6
Intermediate male
54
3
54
Fragment length (bp)
150
200
250
300
350
400
450
475
500

## Slide 3
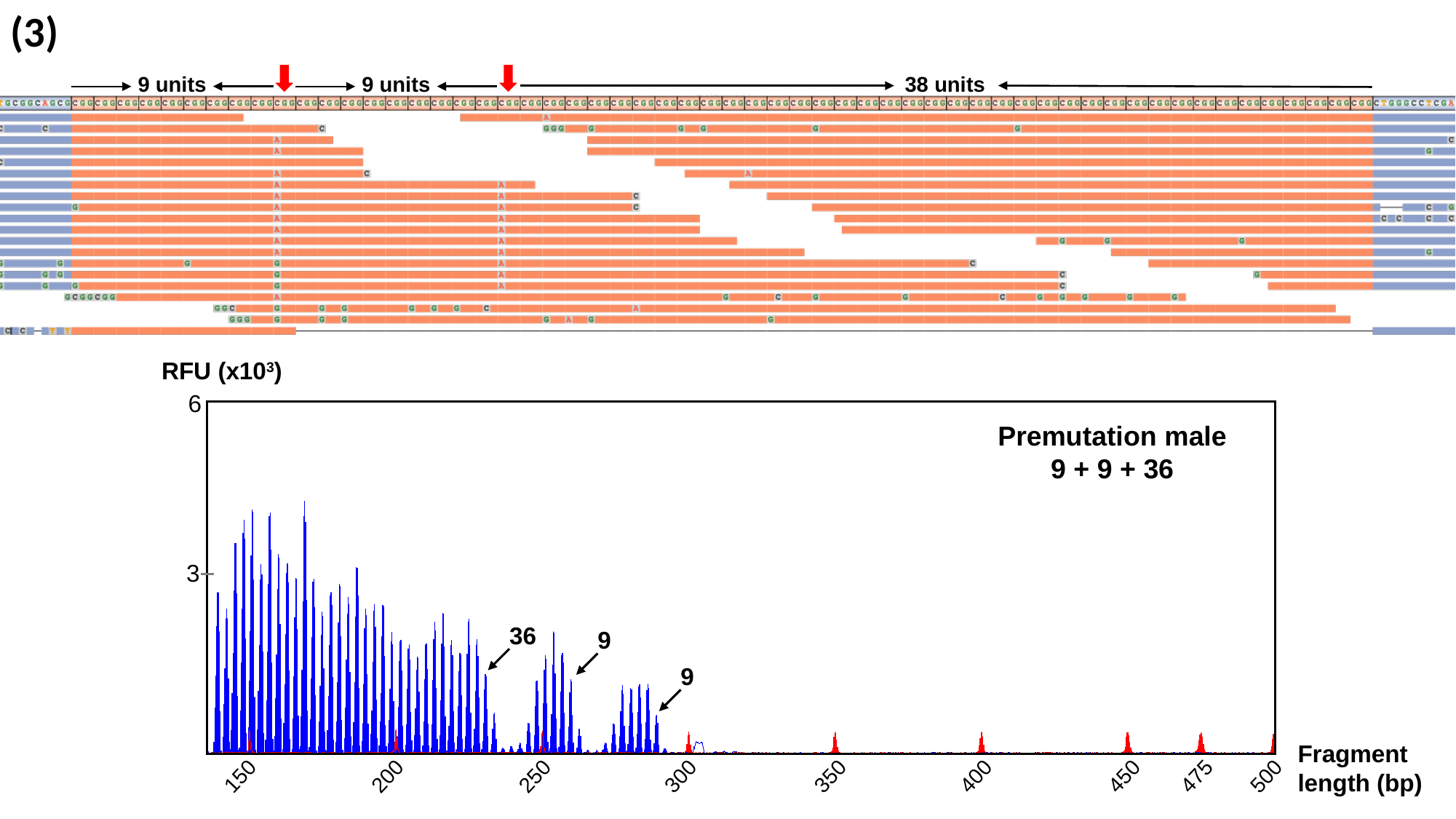

(3)
9 units
9 units
38 units
RFU (x103)
6
Premutation male
9 + 9 + 36
3
36
9
9
Fragment length (bp)
150
200
250
300
350
400
450
475
500

## Slide 4
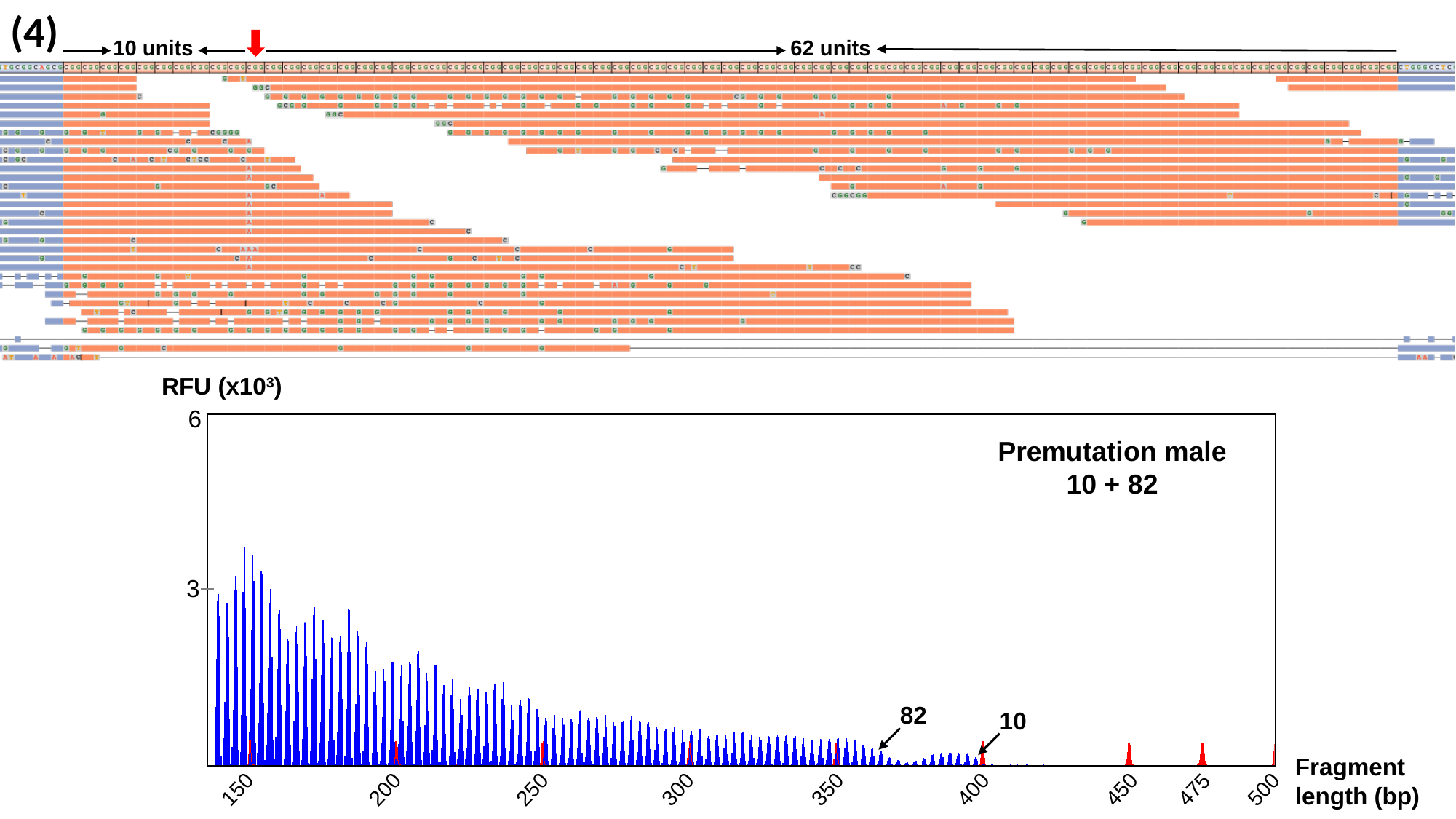

(4)
10 units
62 units
RFU (x103)
6
Premutation male
10 + 82
3
82
10
Fragment length (bp)
150
200
250
300
350
400
450
475
500

## Slide 5
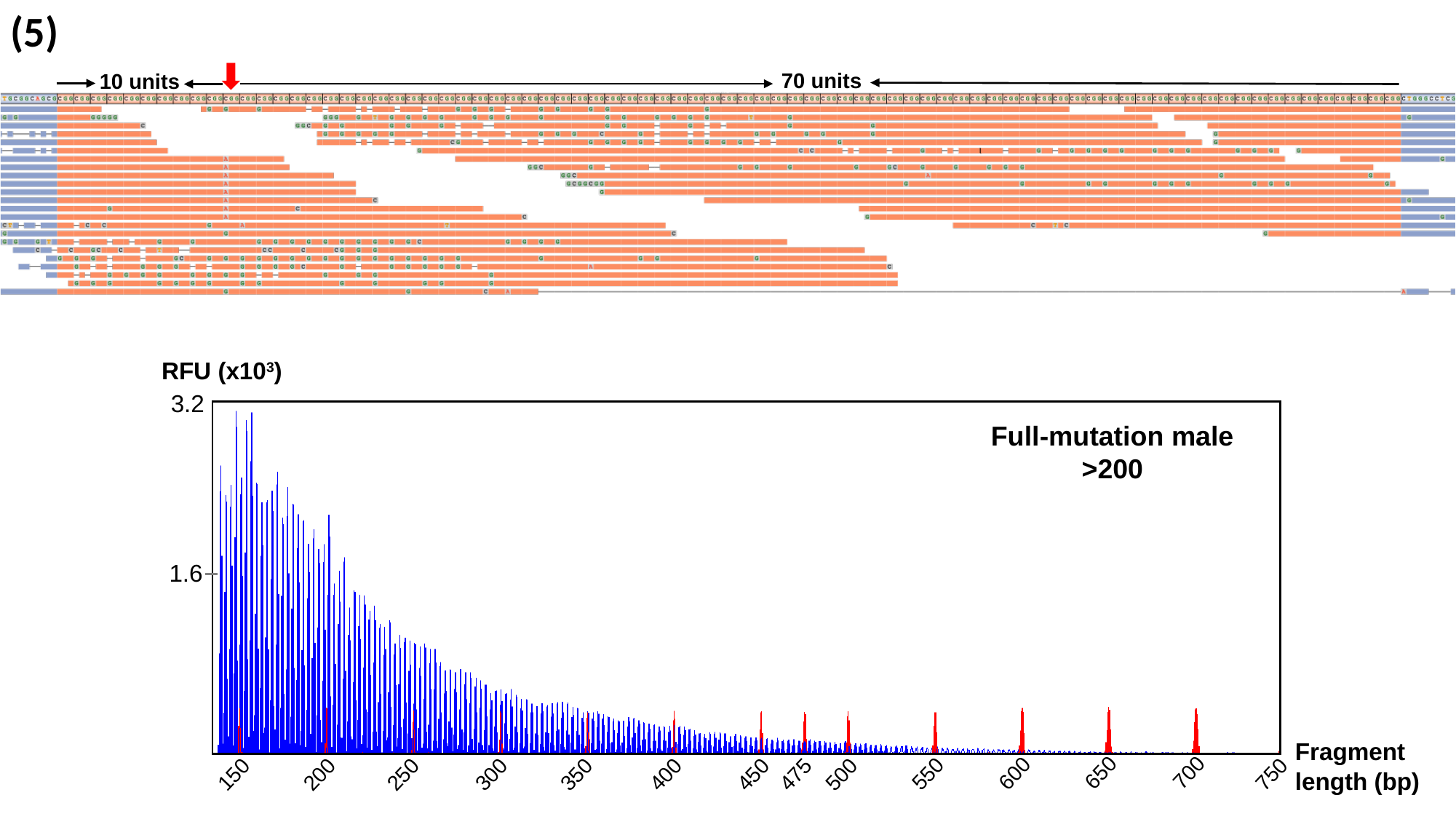

(5)
70 units
10 units
RFU (x103)
3.2
Full-mutation male
>200
1.6
Fragment length (bp)
150
200
250
300
350
400
450
475
500
650
700
600
550
750

## Slide 6
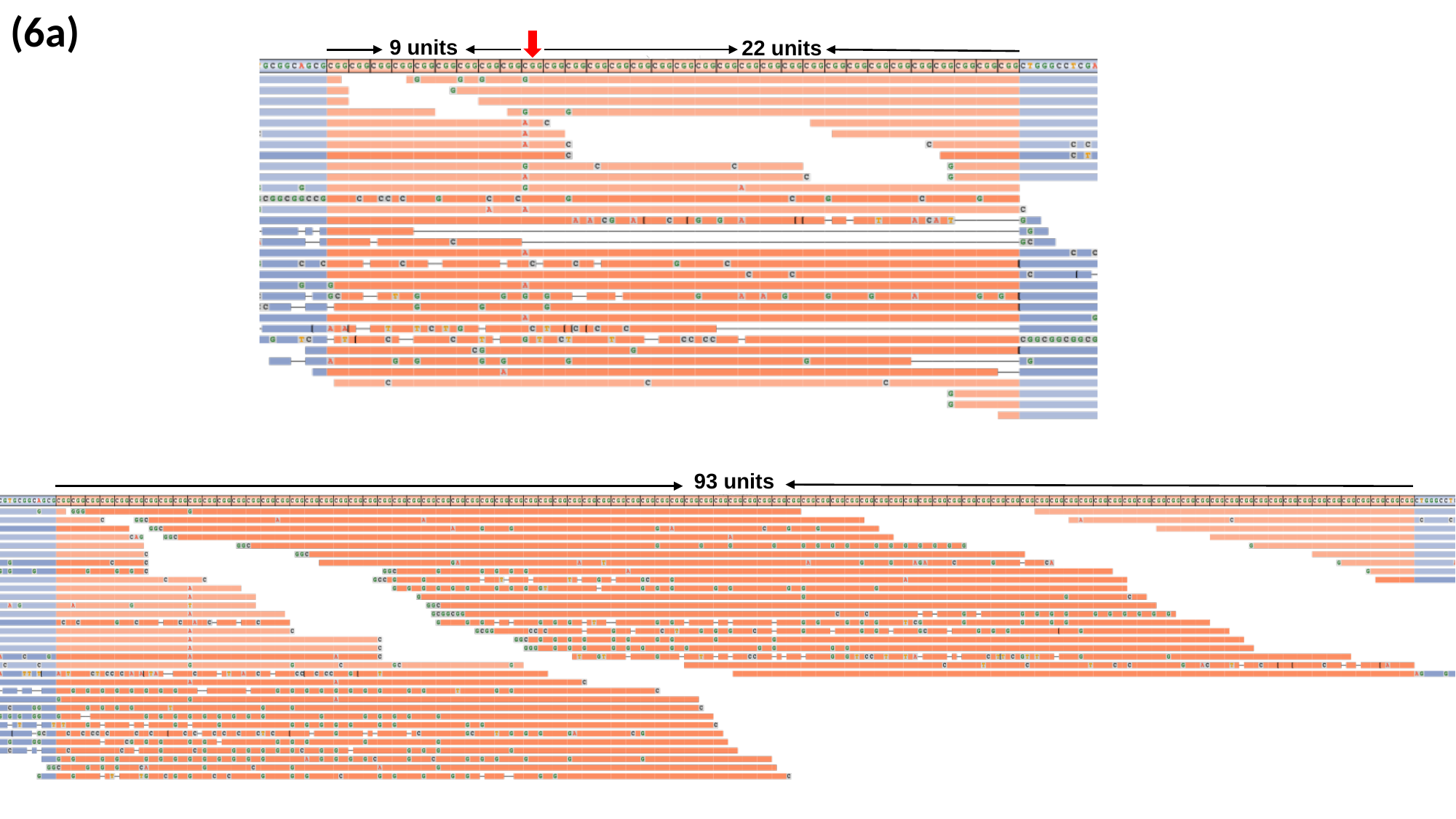

(6a)
9 units
22 units
93 units

## Slide 7
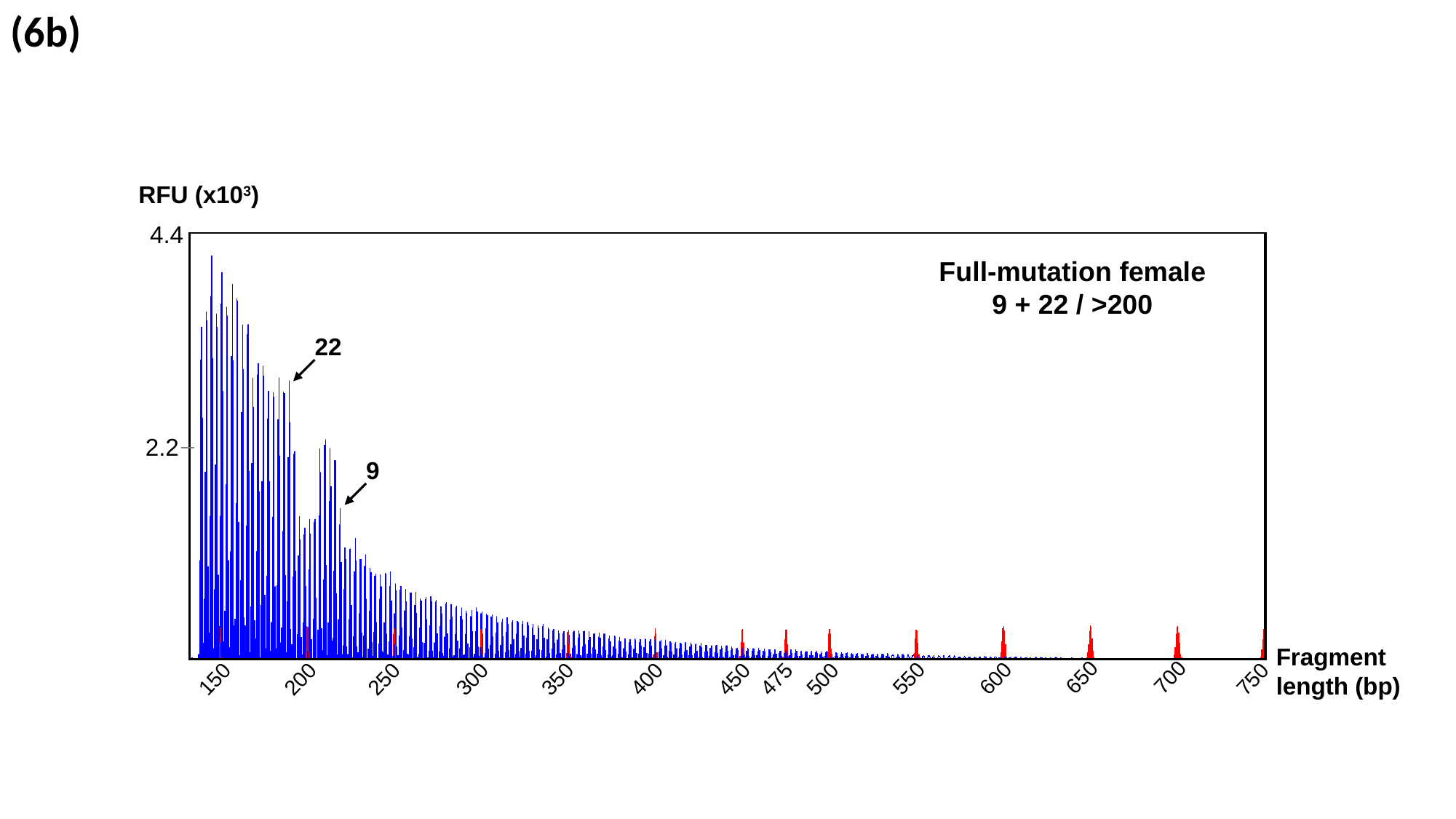

(6b)
RFU (x103)
4.4
Full-mutation female
9 + 22 / >200
22
2.2
9
Fragment length (bp)
150
200
250
300
350
400
450
475
500
650
700
600
550
750
