# Supplementary figures and images for: REViewer: haplotype-resolved visualization of read alignments in and around tandem repeats
Source: Genome Med. 2022 Aug 11;14:84. doi: 10.1186/s13073-022-01085-z (PMC9367089; doi:10.1186/s13073-022-01085-z)

**Fig S1:** REViewer pileup plots for some NA12878 STRs: (A) ATXN10, (B) RFC1, (C) CNPB

**(A) ATXN10 STR**

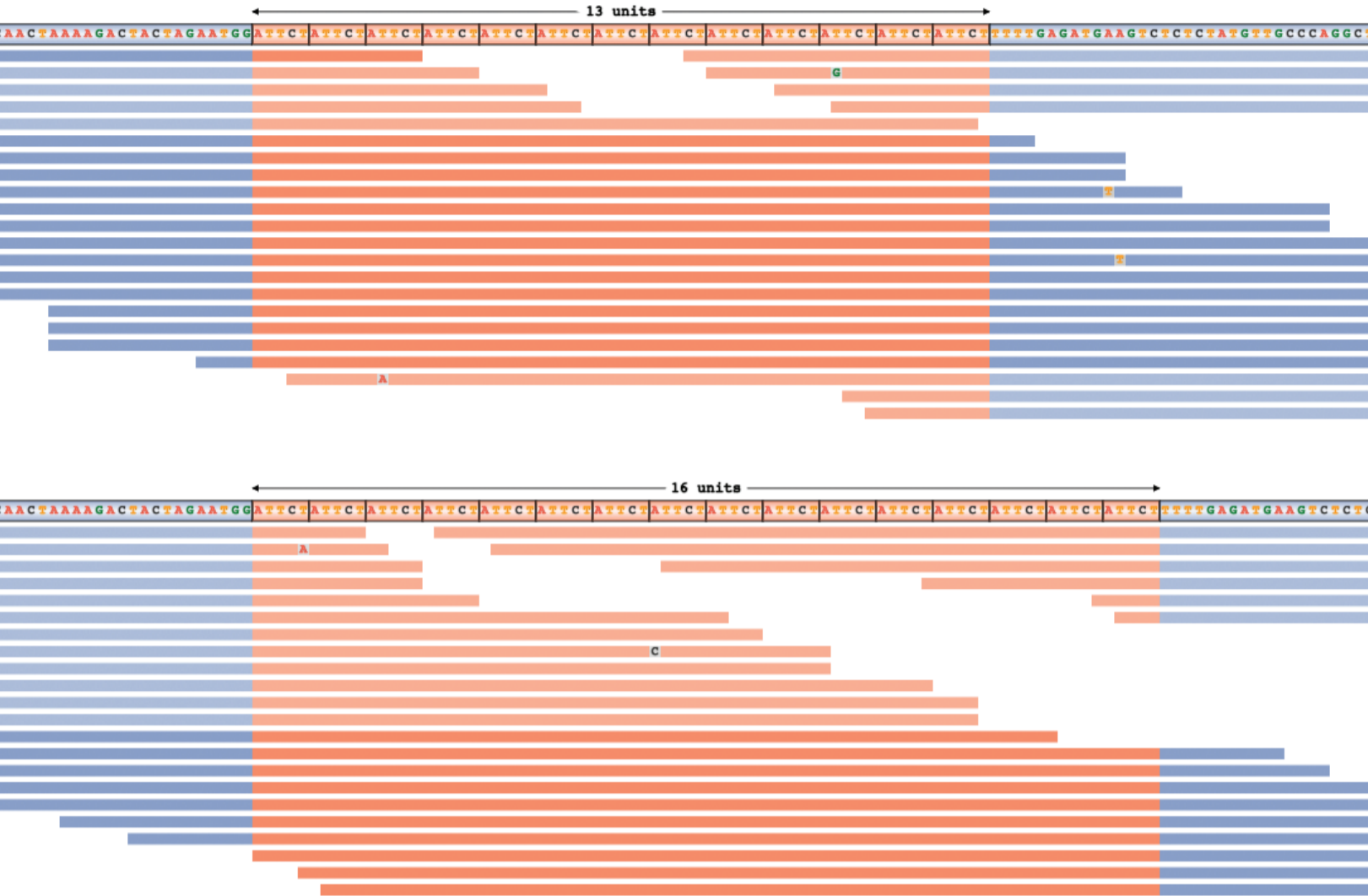

**(B) RFC1 STR**

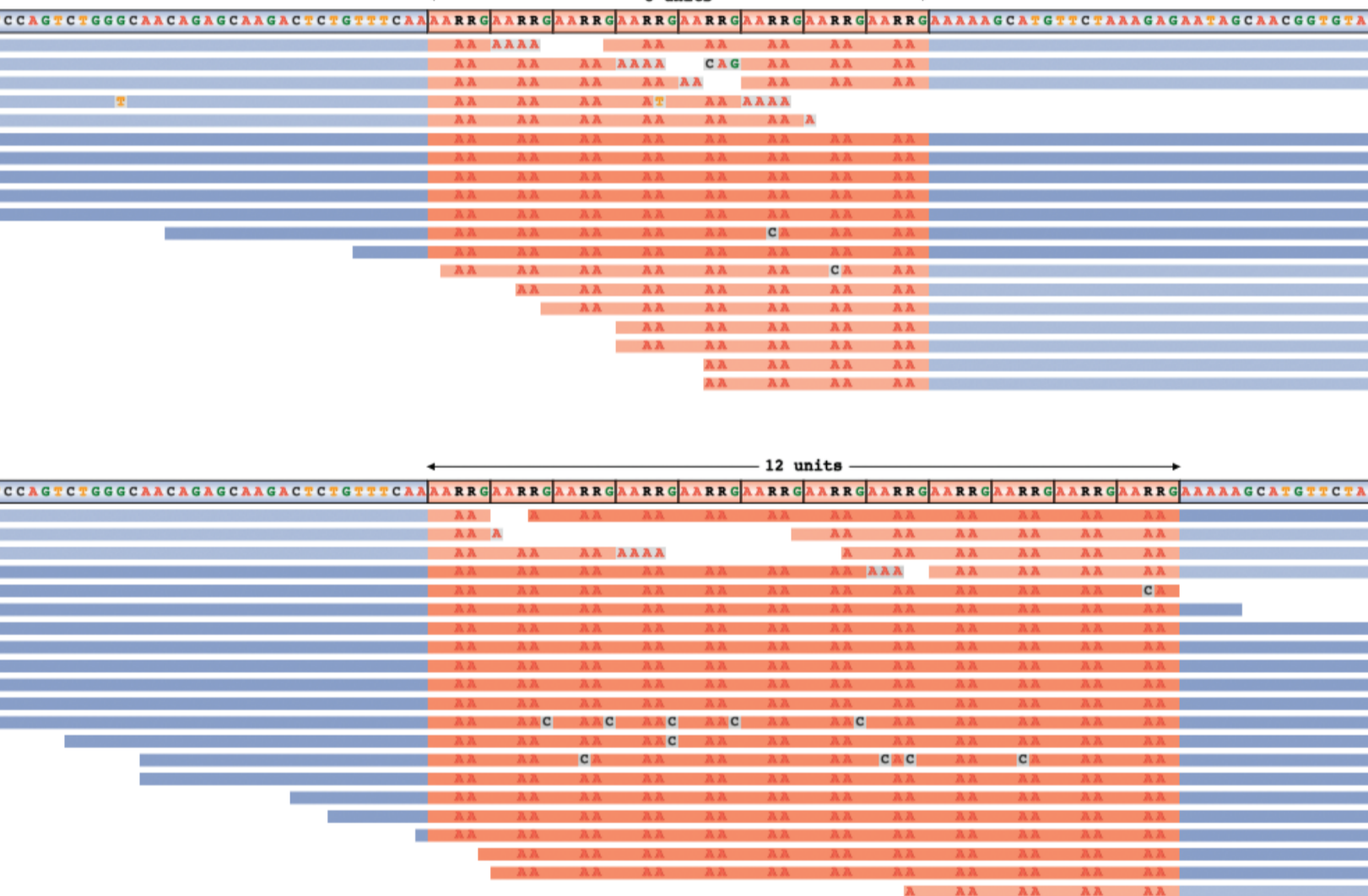

**(C) CNPB STR locus**

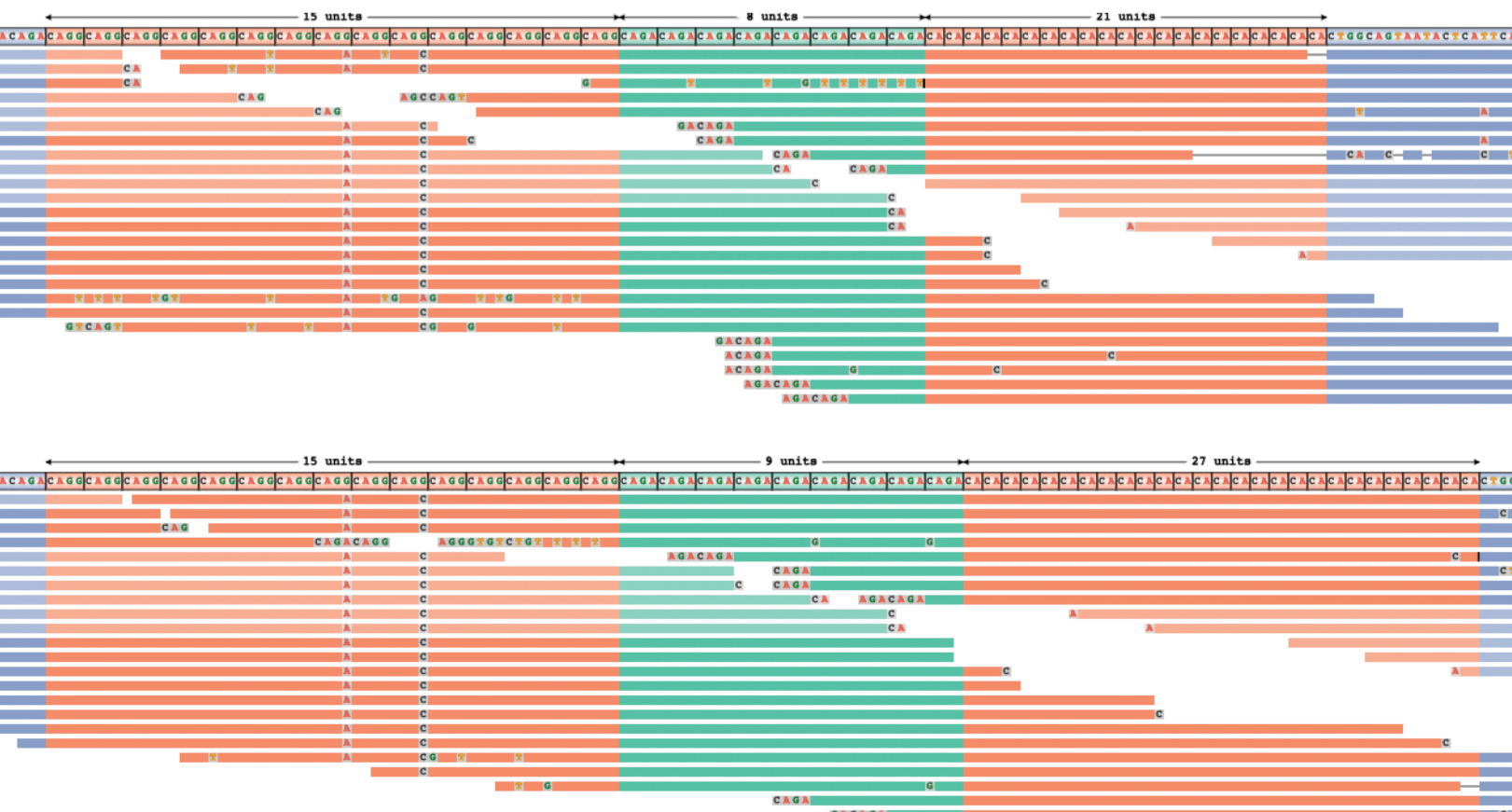

Supplement: Supplementary file 4 — Additional file 4: Figure S1. REViewer pileup plots for some NA12878 STRs: (A) ATXN10, (B) RFC1, (C) CNPB. [file 13073_2022_1085_MOESM4_ESM.pdf]

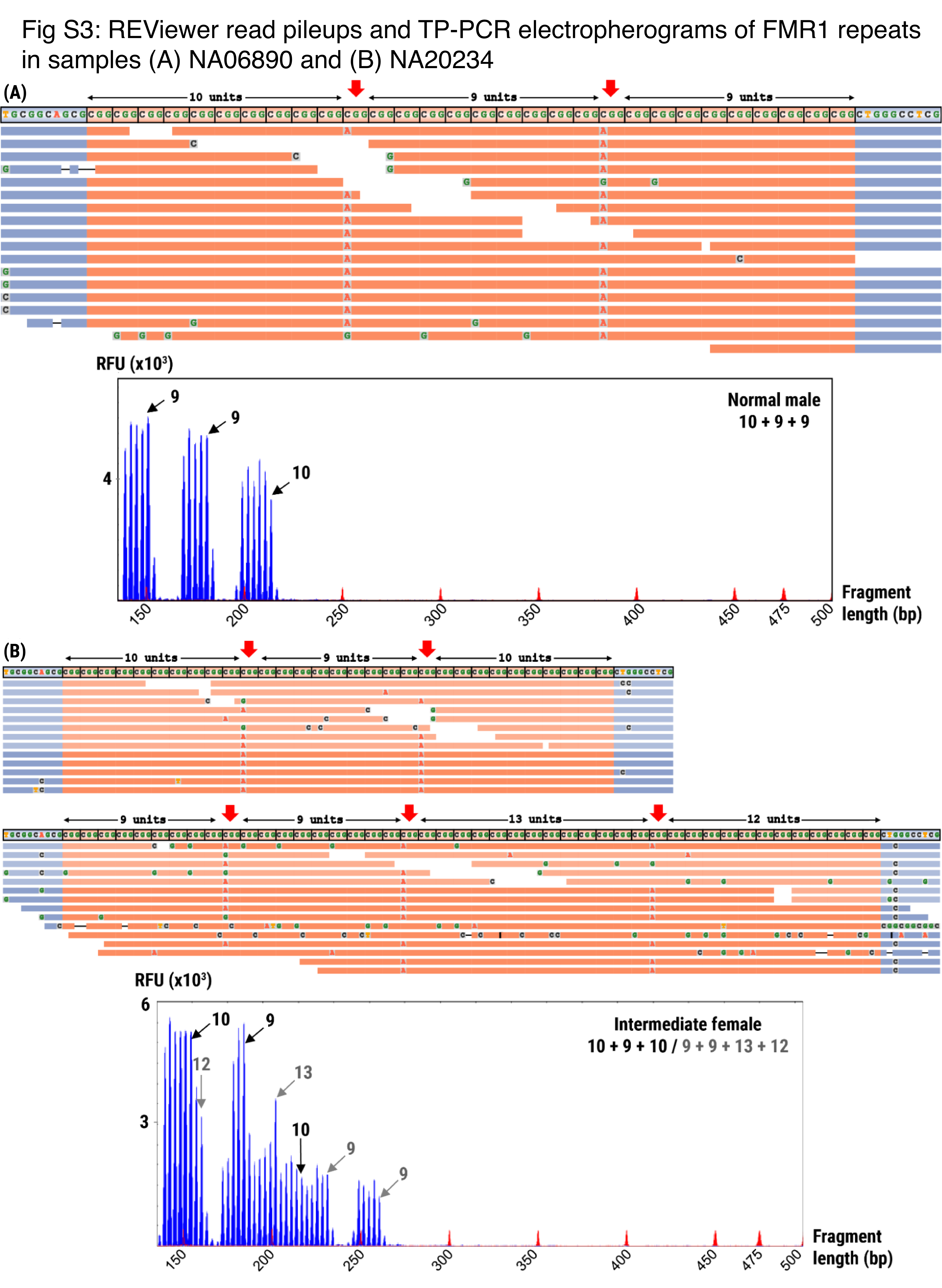

Supplement: Supplementary file 6 — Additional file 6: Figure S3. REViewer read pileups and TP-PCR electropherograms of FMR1 repeats in samples (A) NA06890 and (B) NA20234 [file 13073_2022_1085_MOESM6_ESM.png]
