# Supplementary material for: REViewer: haplotype-resolved visualization of read alignments in and around tandem repeats
Source: Genome Med. 2022 Aug 11;14:84. doi: 10.1186/s13073-022-01085-z (PMC9367089; doi:10.1186/s13073-022-01085-z)

(A) Fig S2: Read pileups in a region surrounding DMPK repeat expansion generated by (A) JBrowse, (B) IGV, and (C) REViewer

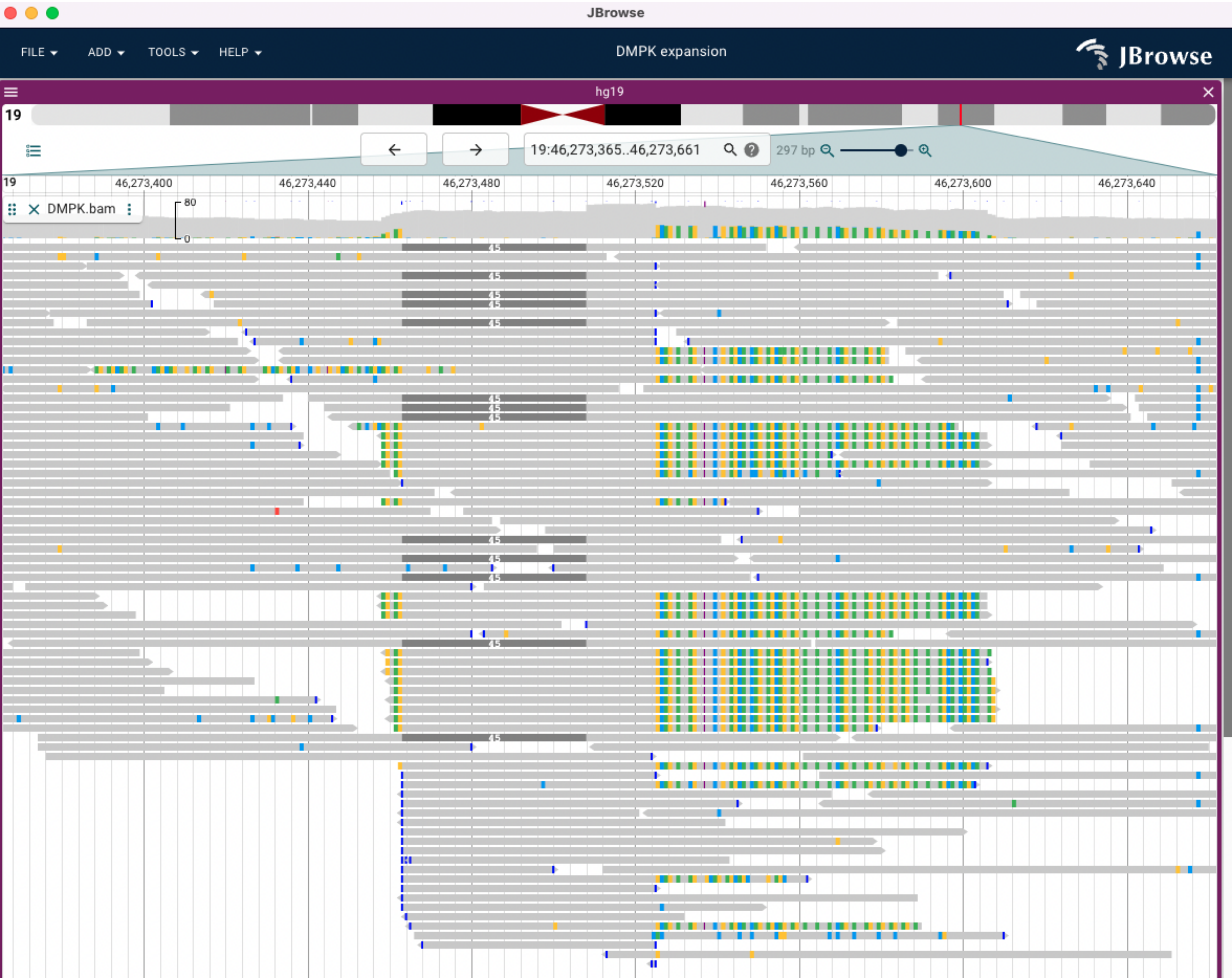

(B)

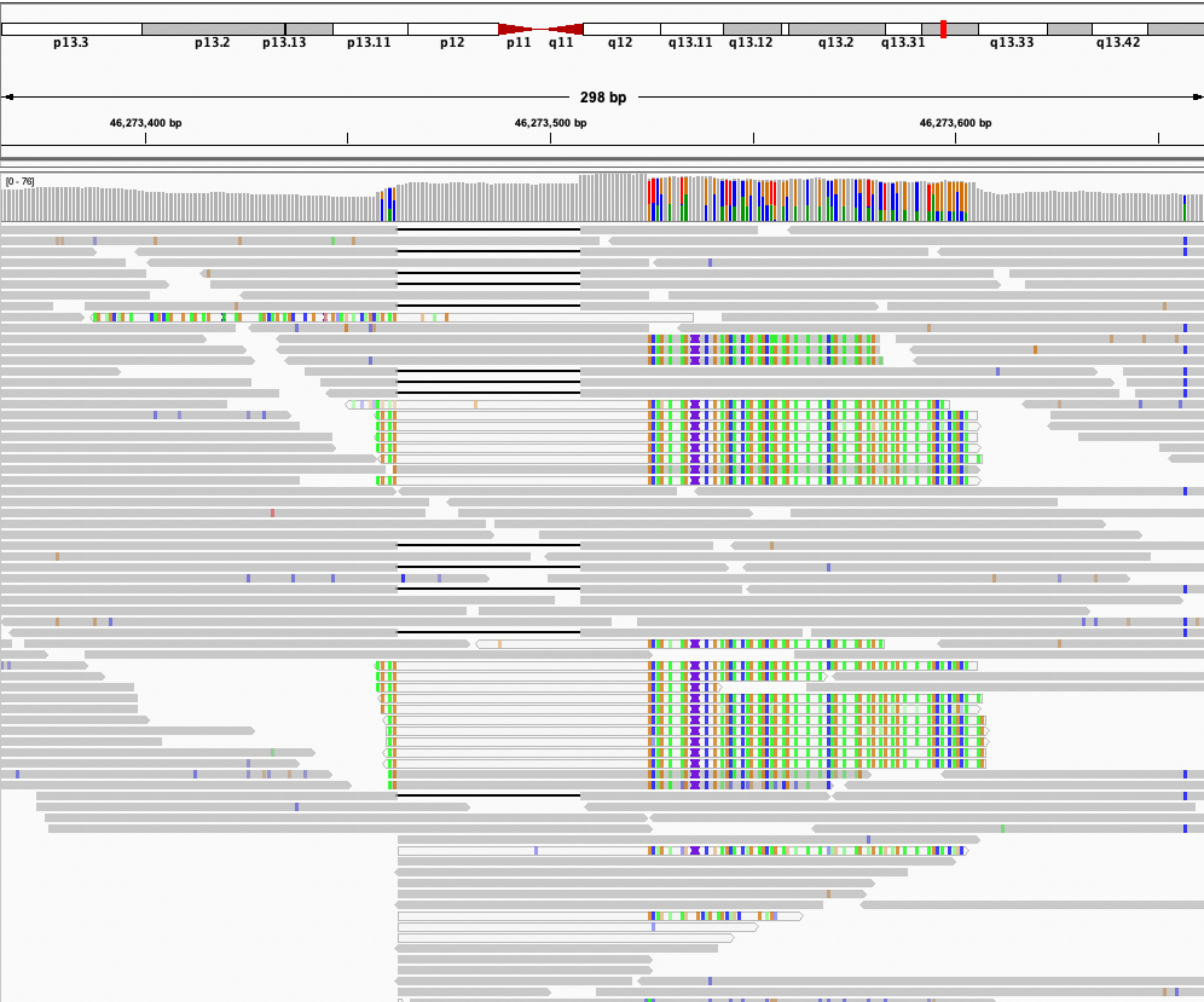
p13.3
p13.2
p13.13
p13.11
p12
p11
q11
q12
q13.11
q13.12
q13.2
q13.31
q13.33
q13.42
298 bp
46,273,400 bp
46,273,500 bp
46,273,600 bp
[0 - 76]

(C)

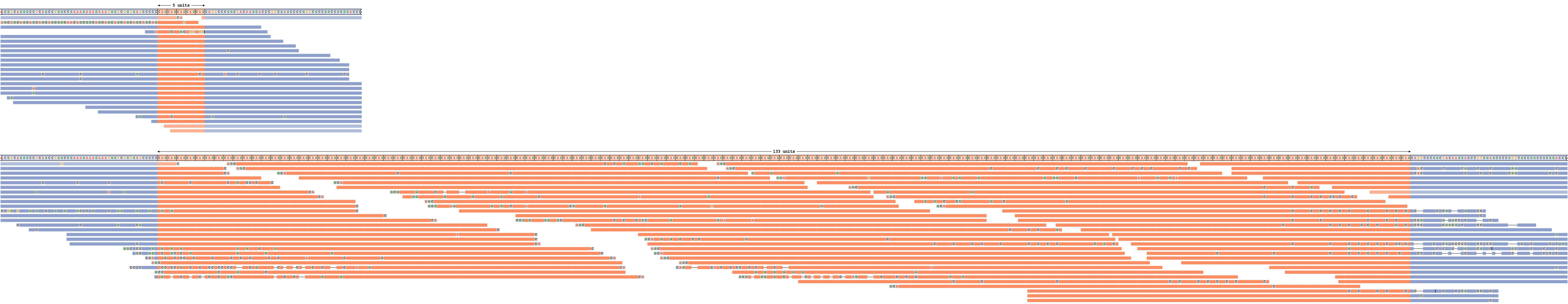

Supplement: Supplementary file 5 — Additional file 5: Figure S2. Read pileups in a region surrounding DMPK repeat expansion generated by (A) JBrowse, (B) IGV, and (C) REViewer. [file 13073_2022_1085_MOESM5_ESM.pdf]
